# Tracheal Pleomorphic Adenoma With Severe Airway Obstruction

**DOI:** 10.7759/cureus.76341

**Published:** 2024-12-24

**Authors:** Natsumi Kushima, Daisuke Himeji, Toyoshi Yanagihara, Kazunari Maekawa, Kousuke Marutsuka

**Affiliations:** 1 Department of Respiratory Medicine, Fukuoka University Hospital, Fukuoka, JPN; 2 Department of Internal Medicine, Miyazaki Prefectural Miyazaki Hospital, Miyazaki, JPN; 3 Department of Diagnostic Pathology, Miyazaki Prefectural Miyazaki Hospital, Miyazaki, JPN

**Keywords:** airway obstruction, bronchoscopy, interventional bronchoscopy, pleomorphic adenoma, tracheal tumor

## Abstract

Pleomorphic adenoma of the trachea is a rare benign tumor, often challenging to diagnose due to nonspecific symptoms. We report a case of a 72-year-old female with a 10-year history of presumed bronchial asthma, presenting with persistent dyspnea. Preoperative assessment for breast cancer surgery revealed severe obstructive ventilatory impairment. Further investigation with chest CT and bronchoscopy identified an intratracheal nodule, which was successfully resected using electrocautery and cryotherapy via rigid bronchoscopy. Pathological examination confirmed pleomorphic adenoma. Following the intervention, the patient’s lung function significantly improved, enabling the planned breast cancer surgery. This case highlights the importance of considering rare tracheal tumors in the differential diagnosis of refractory respiratory symptoms. A review of 11 cases of tracheal pleomorphic adenomas managed by bronchial intervention showed various endoscopic resection techniques, with electrosurgical snaring and argon plasma coagulation being the most common. Our case illustrates the effectiveness of bronchial intervention in managing tracheal tumors with severe airway obstruction and emphasizes the need for thorough preoperative assessment and heightened suspicion for rare tracheal tumors in persistent, treatment-resistant respiratory symptoms.

## Introduction

Tracheal tumors are uncommon, accounting for less than 0.1% of all neoplasms [1]. Pleomorphic adenoma is particularly rare in this location. It typically arises in major or minor salivary glands but can occasionally develop in the palate, lips, nasal area, eyes, cheeks, and ear canal [2]. Composed of epithelial cells, myoepithelial cells, and mesenchymal components within a fibrous capsule, pleomorphic adenoma commonly appears as a slow-growing, painless, well-defined swelling [2]. However, tracheal pleomorphic adenoma presents unique diagnostic challenges due to its nonspecific symptoms and unusual location, often leading to a misdiagnosis of asthma [3].

Fewer than 50 cases of tracheal pleomorphic adenoma have been reported in medical literature to date [4]. We contribute to this limited knowledge by reporting a rare case of tracheal pleomorphic adenoma. Our patient was misdiagnosed with chronic asthma for years before the correct diagnosis was made. After accurate identification, the tumor was successfully treated with bronchoscopic interventions.

## Case presentation

A 72-year-old female presented to our hospital with persistent dyspnea. Ten years earlier, she had been diagnosed with bronchial asthma but discontinued treatment due to poor response. Her medical history included hypertension, sinusitis, osteoporosis, and a left obturator hernia. She was a former smoker with a 30-pack-year history and had worked in a delicatessen.

The patient was initially referred to our surgical department for breast cancer evaluation and diagnosed with left breast cancer (cT1bN0M0; clinical stage I). During the preoperative assessment, pulmonary function tests revealed severe obstructive ventilatory impairment, prompting a referral to our respirology department.

**Figure 1:**
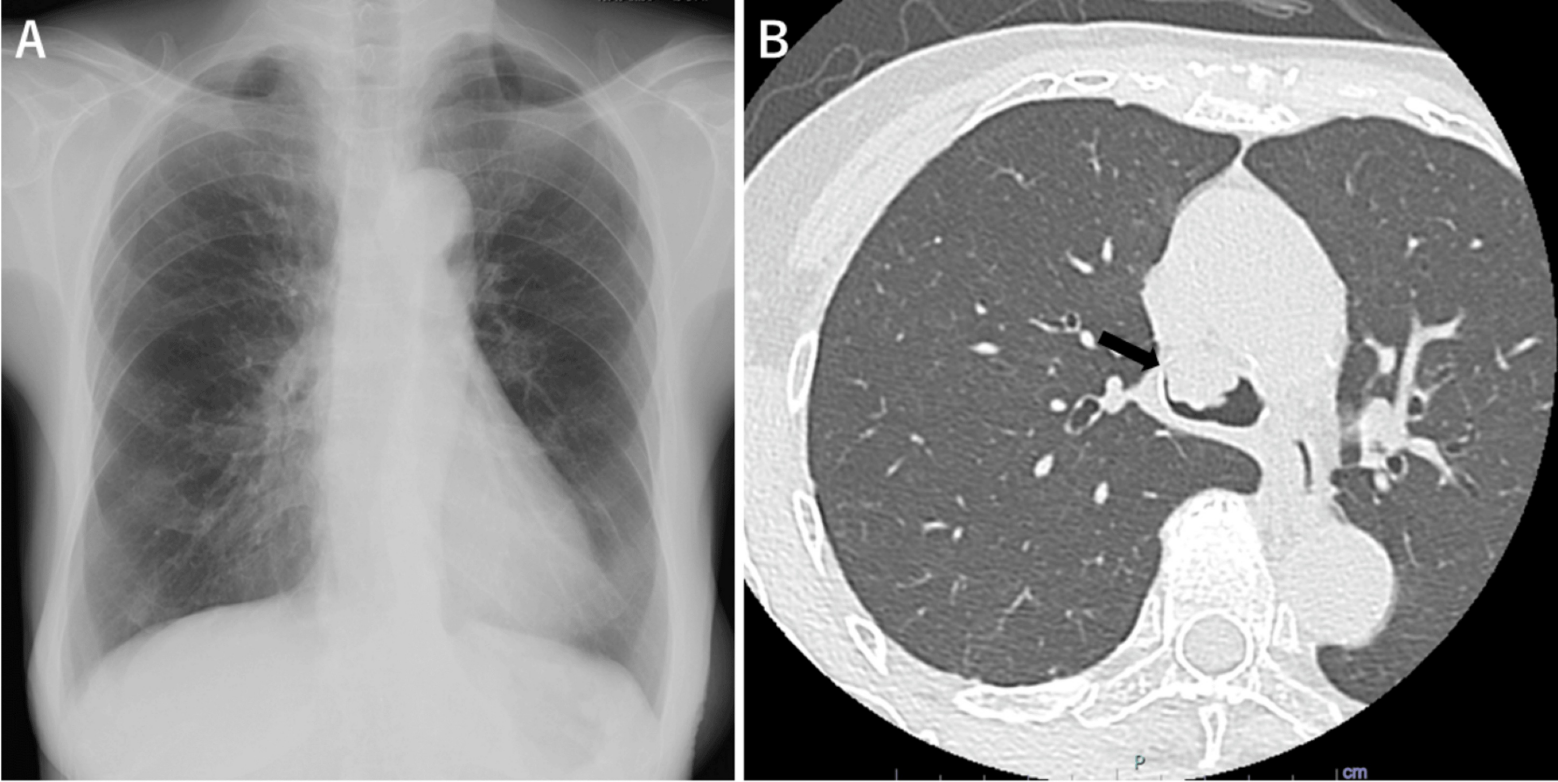
Chest images of the patient (A) Chest X-ray showing no apparent abnormalities in the lung fields or mediastinum. (B) Axial chest CT image showing an intratracheal tumor (black arrow).

On examination, her vital signs were as follows: temperature 36.0°C, heart rate 98 bpm, blood pressure 158/97 mmHg, respiratory rate 18/minute, and SpO2 98% on room air. Physical examination was unremarkable, with clear breath sounds bilaterally and no adventitious sounds. Initial chest X-ray showed no remarkable abnormalities (Figure 1A).

Given her smoking history and obstructive ventilatory impairment, she was treated with a combination of inhaled corticosteroids, long-acting muscarinic antagonists, and long-acting beta-agonists; however, her condition showed minimal improvement.

**Figure 2:**
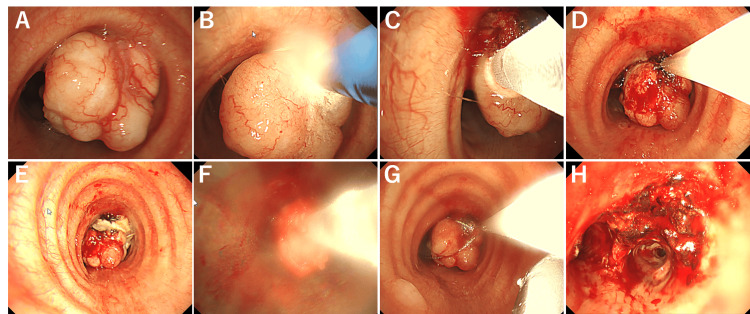
Bronchoscopic images of the step-by-step resection of the tracheal tumor (A) Pre-treatment view of the polypoid tracheal tumor with a smooth, vascularized surface obstructing a significant portion of the airway lumen. (B) Application of argon plasma coagulation (APC). (C) Cryoablation is done with a cryoprobe in contact with the tumor tissue. (D) Tumor resection using an electrosurgical snare, which is looped around the base of the tumor. (E) View of the trachea immediately after tumor resection, showing the residual base of the tumor and the newly opened airway lumen. (F) Retrieval of tumor fragments using the cryoprobe. (G) Additional tumor retrieval using the snare to remove any remaining fragments. (H) After resection.

Further investigation with chest CT unexpectedly revealed an intratracheal nodule (Figure 1B). Subsequent bronchoscopy identified a localized polypoid lesion in the trachea with smooth, transparent epithelium (Figure 2A).

The lesion’s clear margins and lack of disruption to surrounding structures suggested an origin in the epithelial or subepithelial layer.

**Figure 3:**
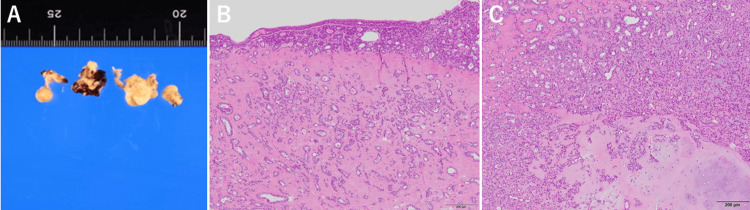
Histopathological findings of the resected tracheal tumor (A) Fragment of removed tumor. The tumor was large and had a slippery surface, so it was removed in sections. (B,C) Hematoxylin and eosin (H&E) staining showing. (B) The surface of this polypoid lesion is covered by nonneoplastic ciliated columnar epithelial cells. (C) The tumor consists of gland-forming epithelial cells, showing a double layer with basal cells, and spindle- or stellate-shaped cells with abundant myxoid or chondroid stroma. These tumor cells are irregularly admixed with each other. No apparent cellular atypism is noted.

Under general anesthesia, we performed bronchial intervention using a DUMON rigidscope (Harada Corporation, Osaka, Japan) (outer diameter 12 mm, inner diameter 11 mm) to secure the airway. The tracheal tumor was resected using a flexible bronchoscope 1TH1200 (Olympus, Tokyo, Japan) with a high-frequency rotatable snare (No. 6183; Boston Scientific, Marlborough, MA), argon plasma coagulation (APC) probe (No. 20132-221; Erbe Elektromedizin GmbH, Tübingen, Germany), electrosurgical system (VIOD300D; No. 10140-100; Erbe Elektromedizin GmbH, Tübingen, Germany), 2.4 mm cryoprobe (No. 20402-411; Erbe Elektromedizin GmbH, Tübingen, Germany), and CRYO2 system (Erbe Elektromedizin GmbH, Tübingen, Germany) (Figure 2B‑Figure 2H). We thought that electrosurgical snaring was better to remove, such as a pedunculated large tumor, but because the tumor seemed to be abundant in blood vessels. Thus, at first, we cauterized the surface of the tumor with an APC probe to manage potential bleeding. Then, we resected the tumor by electrosurgical snaring and removed the slippery tumor from the body by cryoprobe. Pathological examination confirmed pleomorphic adenoma (Figure 3).

**Figure 4:**
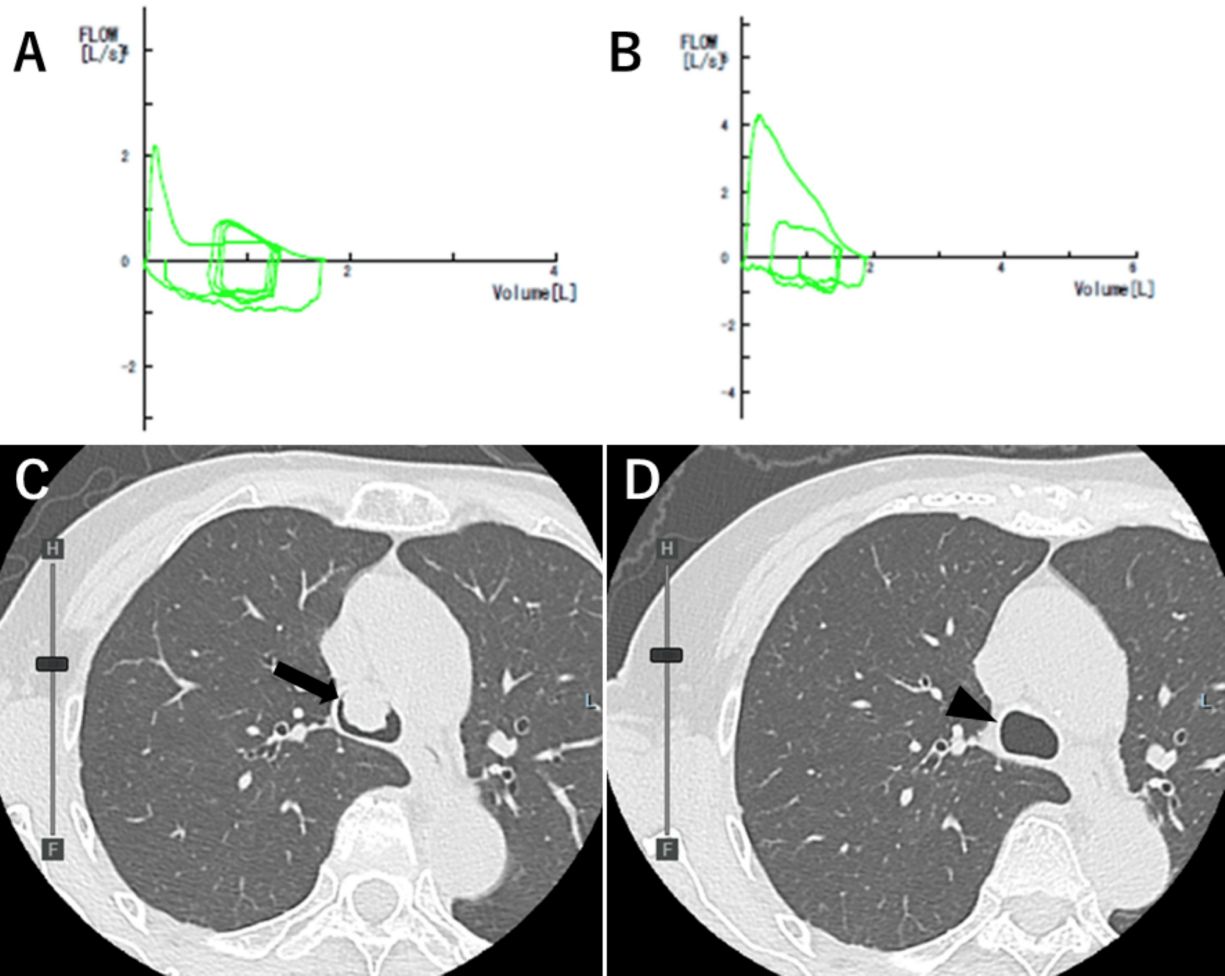
Pulmonary function and images before and after tracheal tumor removal (A) Pre-treatment flow-volume curve showing severe obstruction, with a flattened expiratory limb indicative of fixed upper airway obstruction. (B) Post-treatment flow-volume curve demonstrating significant improvement in airflow, with a more normal curve shape and increased peak expiratory flow rate. (C) Pre-treatment and (D) four months after treatment of chest CT images. The large tumor obstructing most of the trachea (black arrow) was removed (black triangle).

Postoperatively, the patient’s lung function improved significantly, with forced expiratory volume in one second (FEV1) increasing from 0.58 L (35.6% of predicted) to 1.68L (103% of predicted) and peak expiratory flow improving 130 L/minute (33.3% of predicted) to 256 L/minute (64.6% of predicted) (Figure 4A and Figure 4B).

This improvement allowed her to proceed with the planned breast cancer surgery one and a half months after tumor resection. A follow-up chest CT four months post-resection showed no signs of tracheal recurrence (Figure 4C and Figure 4D).

## Discussion

Pleomorphic adenoma is a benign mixed tumor composed of three components: epithelial, myoepithelial, and mesenchymal elements [2]. Primarily associated with salivary glands, its occurrence in the lungs, trachea, and bronchi is extremely rare, accounting for only 2% to 9% of all cases [5]. A comprehensive 34-year, single-institution study of 164 primary salivary gland-type tumors in the tracheobronchial tree highlights this rarity, with pleomorphic adenoma representing just 1.8% of cases, far behind adenoid cystic carcinoma (137 cases, 83.5%) and mucoepidermoid carcinoma (21 cases, 12.8%) [6]. The clinical course of these tumors can be prolonged, with a median symptom duration of 5.5 months; however, cases lasting up to a decade have been reported [4]. Symptoms, often including wheezing, cough, and dyspnea, frequently lead to a misdiagnosis of bronchial asthma. This was evident in our case, where the patient’s symptoms were initially attributed to asthma for an extended period. This case underscores the need to consider rare tracheal tumors in the differential diagnosis of persistent respiratory symptoms, especially when conventional treatments prove ineffective.

Management of tracheal pleomorphic adenoma primarily involves complete surgical resection. However, endoscopic resection using techniques such as APC and electrocautery also has been successful. A review of 11 cases, including ours, of tracheal pleomorphic adenomas managed through bronchial interventions is summarized in Table 1 [3-5,7-13].

**Table 1:** Summary of bronchial intervention for tracheal pleomorphic adenomas APC, argon plasma coagulation

Reference	Year	Age	Sex	Tumor size (cm)	Tumor site	Resection method	Clinical follow-up period (months)	Outcome
Matsubara et al. [7]	2008	71	M	Not available	Left main bronchus	Electrosurgical snaring and APC	Six	No recurrence
Fitchett et al. [5]	2008	65	M	1.3	Right main bronchus	Diathermy snare	Not available	Not available
Kajikawa et al. [8]	2010	55	M	Not available	Lower trachea	APC, electrocautery and rigid bronchoscopic coring	Seven	No recurrence
Goto et al. [9]	2011	71	M	2.5 × 2	Left main bronchus	Electrosurgical snaring	Two	No recurrence
Casillas-Enriquez et al. [10]	2014	33	F	80% occlusion	Upper trachea	APC	Eight	No recurrence
Sim et al. [13]	2014	32	F	1.8 × 1.6	Lower trachea	Rigid forceps and APC	One	No recurrence
Zhu et al. [11]	2018	38	F	1.42 × 0.96	Right main bronchus	Electrosurgical snaring and APC	Three	No recurrence
Takahashi et al. [3]	2019	51	F	1.5	Upper trachea	Electrosurgical snaring and forceps	30	No recurrence
David et al. [12]	2020	83	F	1.6 × 1.3	Upper trachea	Fiber-based CO_2_ laser and rigid bronchoscope	Not available	Not available
Liao et al. [4]	2020	49	F	2.4 × 2.1	Lower trachea	Electrosurgical snare, cryotherapy, and APC	Three	No recurrence
Our case	2023	72	F	1.5 × 1.2 × 1.7	Lower trachea	Electrosurgical snare, APC, cryotherapy, and rigid bronchoscope	15	No recurrence

Patient ages ranged from 32 to 83 years (median 55), with a slight female predominance in recent cases. More than half of the cases had a previous diagnosis of obstructive lung disease (data not shown). Tumors varied in location throughout the tracheobronchial tree, ranging in size from 1.3 cm to 2.5 × 2 cm, with our case measuring 1.5 × 1.2 × 1.7 cm. In the literature presented in Table 1, although some reports did not clearly document the reasons for choosing endoscopic resection over surgical resection, bronchoscopic resection was selected for reasons such as patient preference against surgery [7], the tracheal tumor was smaller than the tracheal diameter, and there was thought to be no risk of tracheal obstruction due to resection [3] and the procedure being less invasive with faster postoperative recovery [4]. We believe that surgical resection is the preferred treatment for tracheal tumors when a definitive diagnosis has been made, the respiratory status is stable, and the extent of resection is feasible. However, in cases where airway stenosis caused by the tumor at the time of presentation was severe, transbronchial biopsy for definitive diagnosis will be risky for those patients. In such cases, we believe that it will be better to first secure the airway endoscopically and then consider additional treatments, such as surgical resection, based on the degree of curability with endoscopic treatment, the results of definitive diagnosis, and an assessment of surgical resectability.

Various endoscopic resection techniques were used, often in combination. Electrosurgical snaring was the most common (seven of 11 cases), frequently complemented with APC (six cases). Other methods included rigid bronchoscopy, forceps, cryotherapy, and, in one instance, fiber-based CO_2_ laser. We use a rigid bronchoscope to secure the airway. We resected the tumor with an electrosurgical snaring and managed to bleed with APC, as in the majority of the other cases in Table 1. A cryoprobe was used for removing from the body through rigidscope. In our case, bronchial intervention proved effective, allowing for tumor removal while preserving tracheal function.

In cases of incomplete resection, recurrence and malignant transformation are possible [14]. There are few reports of long-term follow-up after excisional treatment of tracheal pleomorphic adenoma, so the rate and timing of recurrence are unknown. A case of tracheal pleomorphic adenoma was reported as recurrent in 2020, following surgical resection and end-to-end anastomosis performed 10 years earlier [15]. We would like to follow this patient by periodic chest CT and flexible bronchoscopy at least 10 years after the tumor resection.

This case illustrates several key points. First, it emphasizes the importance of thorough preoperative assessment, including imaging studies, in patients with persistent respiratory symptoms. Second, it highlights the need for a high index of suspicion for rare tracheal tumors in cases of refractory "asthma." Last, it demonstrates the effectiveness of bronchial intervention techniques in managing tracheal tumors, even in cases with severe airway obstruction.

## Conclusions

While pleomorphic adenoma commonly occurs in salivary glands, its occurrence in the trachea is exceptionally rare. The tumor occurring in the trachea typically presents with obstructive airway symptoms that can mimic asthma and lead to misdiagnosis. Despite limited consensus on management and prognosis due to its rarity, bronchoscopic tumor resection can be highly effective, as demonstrated in our case. Early recognition and appropriate intervention can lead to favorable outcomes and improved quality of life. Long-term follow-up is essential to monitor for recurrence and potential malignant transformation.
